# Spatiotemporal modeling of under-five mortality and associated risk factors in Ethiopia using 2000–2016 EDHS data

**DOI:** 10.1186/s12887-024-04676-4

**Published:** 2024-03-21

**Authors:** Endashaw Amuka, Aweke A. Mitiku, Melkamu A. Zeru

**Affiliations:** Department of Statistics, College of Science, Bahir Dar University, Bahir Dar, Ethiopia

**Keywords:** Under-five children mortality, Space-time dynamic, Spatiotemporal, Ethiopia

## Abstract

**Background:**

The under-five mortality rate serves as a key indicator of the performance of a country’s healthcare system. Despite a minor decline, Ethiopia continues to face a persistently high under-five mortality rate across different zones. Thus, this study aimed to identify the risk factors of under-five mortality and the spatiotemporal variation in Ethiopian administrative zones.

**Method:**

This study used the 2000–2016 Ethiopian Demographic and Health Survey (EDHS) data which were collected using a two-stage sampling method. A total of 43,029 (10,873 in 2000, 9,861 in 2005, 11,654 in 2011, and 10,641 in 2016) weighted sample under-five child mortality were used. The space-time dynamic model was employed to account for spatial and time effects in 65 administrative zones in Ethiopia.

**Results:**

From the result of a general nesting spatial-temporal dynamic model, there was a significant space-time interaction effect [**γ =** -0.1444, 95% CI(-0.6680, -0.1355)] for under-five mortality. The increase in the percentages of mothers illiteracy [β = 0.4501, 95% CI (0.2442, 0.6559)], not vaccinated[β= 0.7681, 95% CI (0.5683, 0.9678)], unimproved water[β= 0.5801, CI (0.3793, 0.7808)] were increased death rates for under five children while increased percentage of contraceptive use [β= -0.6609, 95% CI (-0.8636, -0.4582)] and antenatal care unit visit > 4 times [β= -0.1585, 95% CI(-0.1812, -0.1357)] were contributed to the decreased under-five mortality rate at the zone in Ethiopia.

**Conclusions:**

Even though the mortality rate for children under five has decreased over time, still there is higher in different zones of Ethiopia. There exists spatial and temporal variation in under-five mortality among zones. Therefore, it is very important to consider spatial neighborhood’s and temporal context when aiming to avoid under-five mortality.

## Background

Under-five child mortality is a leading indicator of child health and the state of a country’s total development as it reflects the social, economic, and environmental conditions that children (and other members of society) live in, including their access to healthcare [1]. It is the likelihood that a child will pass away before reaching five years, represented as a rate for every 1,000 live births [2]. When compared to the anticipated 9.92 million deaths of children aged under five in 2000, the estimated number of children under the age of five worldwide in 2019 was 5.30 million [3]. With this decrease, the under-five child mortality rate has decreased from 75 for every 1,000 live births in 2000 to 38 for every 1,000 live births in 2019. Despite such a remarkable decrease, the worldwide under-five child mortality rate is still significantly higher than the sustainable development goal of 25 fatalities for every 1,000 live births [4].

Sub-Saharan Africa (SSA) nations may have a unique sociocultural setting, and children there may be exposed to a variety of dangers that can be fatal [5]. The baseline indicators for each goal in 2015 differ significantly from one nation to the next and since different areas around the globe do not have the same resources to improve the standard of living of individuals. As a consequence, the indicators used to evaluate how well the 2030 targets are being met will evolve over time differently in each region [6].

Various studies on under-five mortality in Ethiopia have been carried out in the past [7–15]. Nevertheless, these researches primarily examined the risk variables for under-five mortality without considering spatial variation and temporal changes. The spatial pattern and temporal variation of under-five mortality across zones in Ethiopia, thus, are not well covered in the studies. Therefore, it is critical to include the evidence that suggests under-five mortality may become concentrated in a particular location over time in order to evaluate the public health effects of mother and child health intervention programs already implemented [16]. In order to achieve Ethiopia’s growth and transformation strategy hopes to lower the mortality rate for children under the age of five to less than 30 per 1000 live births additional research is important. To address this gap, we conducted an all-inclusive cross-sectional analysis from the recent 2000 to 2016 EDHS to model spatiotemporally for the major risk factors of under-five mortalities in Ethiopia, taking into consideration the space and time effect. Many studies were conducted in Ethiopia about spatial patterns of under-five mortality, yet the temporal variation was not addressed. The main motivation behind spatiotemporal investigation is; that a study allows us to understand the geographical distribution and temporal trends of under-five mortality across different zones and over time. This information helps to identify areas with high mortality rates, enabling targeted interventions and resource allocation to address the disparities. Additionally, a spatiotemporal analysis can provide insights into the underlying factors contributing to under-five mortality. It allows us to examine the social, economic, and environmental determinants specific to different zones, helping policymakers and healthcare providers develop context-specific strategies to reduce child mortality. Moreover, studying the spatiotemporal patterns of under-five mortality helps monitor the progress of interventions and policies implemented to improve child health. By tracking changes over time, researchers and policymakers can assess the effectiveness of various initiatives and make informed decisions on adjustments or scaling up successful interventions. Overall, a spatiotemporal study on under-five child mortality in Ethiopia is crucial for informed decision-making, targeted interventions, and monitoring progress in reducing child mortality rates across the country.

Therefore, the main aim of this study is to investigate the spatiotemporal variation of under-five mortality and associated factors in Ethiopia administrative zones based on the EDHS, 2000–2016 dataset.

## Methods

### Study area and data source

**Fig. 1:**
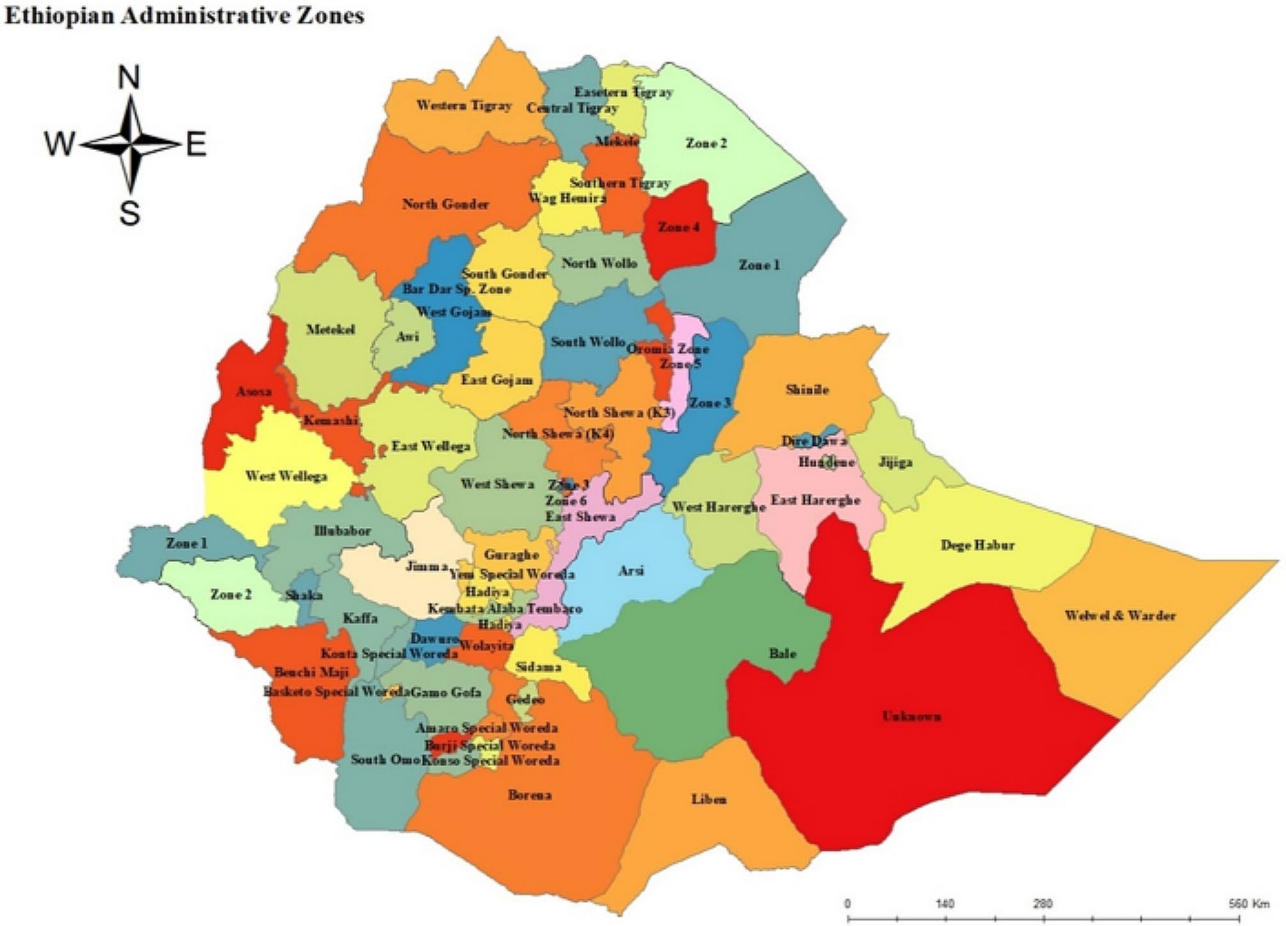
Map of Ethiopia administrative zones

The data was obtained from 65 administrative zones in Ethiopia. Ethiopia shares borders with Eritrea in the north, Djibouti and Somali in the east, Sudan and South Sudan in the west and Kenya in the south. The World Meter estimated that the country had about 120 million people. Administratively, Ethiopia is divided into 11 exclusive units (regions) including Addis Ababa, the capital city of the country and 72 administrative zones as CSA reported in 2013 [17] (Fig. 1).

The data was obtained from the Ethiopian Demographic and Health Survey (EDHS) Programme, which is accessible to many nations, particularly those with low incomes. The DHS data is readily accessible and publically available at https://dhsprogram.com.

To offer the most accurate data on indicators related to maternal and child health, the EDHS data is a series of nationally representative demographic and health surveys conducted in the country every five years. This study took into account four EDHS that were completed in 2000 along with 2005, 2011 and 2016, allowing for a summary of the results for the entire nation. A stratified two-stage cluster sampling procedure was used to select the nationally representative sample in all four surveys. In the first stage, clusters or enumeration areas (EAs) were selected with probability proportional to population size. A total of 540, 540, 624 and 645 EAs (clusters) were selected in 2000, 2005, 2011 and 2016, respectively. The second stage involved the stratified sampling of households in each selected cluster.

The study was conducted on 43,029 children consisting of 10,641 from 2016, 11,654 from 2011, 9861 from 2005, and 10,873 children from the 2000 EDHS respectively.

The outcome variables in this study were the proportion of under-five child mortality (U5M) for the Ethiopian administrative zones. As predictor variables, the following demographic, social, geographic, and environmental factors have been guided by existing literature studying the determinants of children under age five. We utilized the percentage of dependent and independent variables that have binary categories for the spatial and space-time dynamic model. We have collapsed the variables by administrative zones to get a proportion of the variables (Table 1).

In order to do this, we used the geographical variables from the GPS dataset of the demographic and health survey data and connected them to the DHS row dataset. Finally, utilizing both the EDHS and geographical variables, we were able to predict under-five mortality at the zonal level.

**Table 1:** The description of explanatory variables included in the model

Under-five child mortality (Dependent variable) $$yi\, = \,\left\{ {\begin{array}{*{20}{c}}{1\,if\,a\,child\,is\,Died}\\{0\,if\,a\,child\,is\,Alive}\end{array}} \right.$$	
Covariates	Descriptions
% father with illiteracy	The proportion of father with illiteracy
% of unemployment status of women	The proportion of unemployment status of women
% of a child was rural	The proportion of child was from urban
% family with wealth status	The proportion of families with wealth status
% of access to unimproved source water	The proportion of access to unimproved source water
% of unimproved toilet facility	The proportion of unimproved toilet facility
% of women with media exposures	The proportion of women with media exposures
% mother with illiteracy	The proportion of mothers with illiteracy
% of a child was Male	The proportion of children was Male
% family with household sizes > 6	The proportion of families with household sizes > 6
% Age at first marriage < = 16	The proportion of Age at first marriage < = 16
% birth order of child > 3	The proportion of birth order of child > 3
% marital status with married	The proportion of marital status with married
% preceding birth interval in month < = 36	The proportion of preceding birth interval in months < = 36
Average precipitation	The average precipitation with in 10 km(rural) or 2 km(urban)
Average malaria prevalence	The average malaria prevalence in 10 km(rural)or 2 km(urban)
Average max. temperature	The average max. temperature within 10 km(rural) or 2 km(urban)
Average wet days	The average wet days with in 10 km(rural) or 2 km(urban)
% Women with contra use	The proportion of women with contra use
% Children with not currently breastfeeding	The proportion of children with no current breastfeeding
% of delivery within at health center	The proportion of delivery within a health center
% child was not vaccinated	The proportion of child was vaccinated
% ANC visit at least 4 times	The proportion of ANC visits at least 4 times

### Statistical methodology

The traditional linear models computed using ordinary least squares approaches cannot account for the fact that data collected based on spatial and temporal criteria is not independent of its spatial location across different times. If the model ignores the spatial and temporal influences, the estimated values will be distorted. Different model specifications must be considered in order to accommodate different pairings of the two instances. These observations are accessible over time of space N spaces and T time points [18].

Let $\varvec{y_{i}}$ stand for an observation vector on the dependent variable with spatial units (i = 1, 2… N), and temporal units (t = 1, 2… T) of NT × 1 columns. The spatial weight matrix W, which remains constant throughout time, is the N × N positive matrix defining the spatial arrangement of the n units with diagonal elements set to zero. Let X be an NT × k matrix containing observations on the covariates. The spatial weight matrix connected to units i and j is represented by each element 𝑤_ij_ ∈ 𝑾 [18]. The element (i, j), the neighborhood matrix of the row standardized matrix with its dimension $65\times 65$ is the component of 𝑤_ij_. Therefore, the matrix’s non-zero members show whether the two sites are neighbors. This weighted matrix is commonly expressed as:

$${{\rm{w}}_{{\rm{ij}}}}{\rm{ = }}\left\{ {\begin{array}{*{20}{c}}{{\rm{1,}}\,{\rm{if}}\,{\rm{the}}\,{\rm{area}}\,{\rm{i}}\,{\rm{and}}\,{\rm{j}}\,{\rm{are}}\,{\rm{neighboring}}}\\{{\rm{0,}}\,{\rm{otherwise}}}\end{array}} \right.$$

The existence of spatial autocorrelation in the dataset is a semi-variogram. The semi-variogram function γ (h) is defined as half the average squared difference between points separated by a distance ℎ, expressed as:

$$\gamma \left( {\rm{h}} \right) = \frac{1}{{2\left| {{\rm{N}}\left. {\left( {\rm{h}} \right)} \right|} \right.}}\sum\limits_{{\rm{i}} = 1}^{\rm{n}} {{{({{\rm{Z}}_{\rm{i}}} - {{\rm{Z}}_{\rm{j}}})}^2}}$$

where (ℎ) is the set of all pairwise Euclidean distances 𝑖 − 𝑗 = ℎ, | (ℎ)| is the number of distinct pairs in (ℎ), n is the number of observations (polygons) in the whole cluster and Z_i_ and Z_j_ are data values at spatial locations 𝑖 and 𝑗, respectively.

**Fig. 2:**
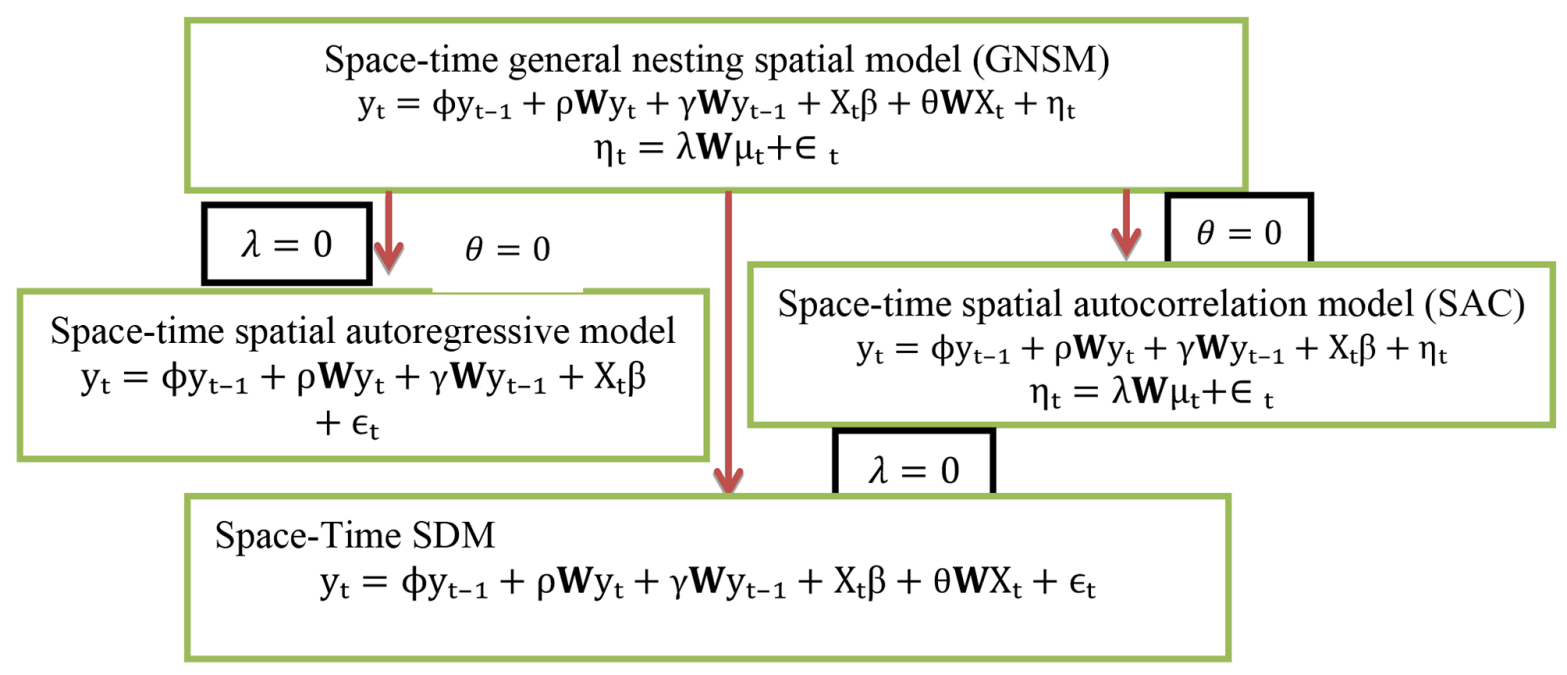
Space-time dynamic regression models

We used the four fundamental models of spatial time dynamics: the spatial Durbin model, the spatial autoregressive model, the spatial error model, and the general nested model with space-time. Let **Wu** and **WX** represent the interactions between the error terms of various observations and the covariates with spatial components, respectively. [𝑾𝒚_t-i_] is the i^th^ element of the spatial lag vector for the same period. The i^th^ element of the spatial lag vector of observations made on the response variable in a prior time is represented by the ${y_{i-1}}$. The model is referred to as a space-time recursive model when the response variable is connected to the same places as well as the nearby locations in another period. The dependent variable’s data from the prior period are represented by $y_{i}$. Let the spatial dependence ρ, spatiotemporal diffusion γ, and autoregressive time ϕ dependence parameters (Fig. 2).

## Results

### Spatial and temporal patterns of under-five child mortality across administrative zones

**Fig. 3:**
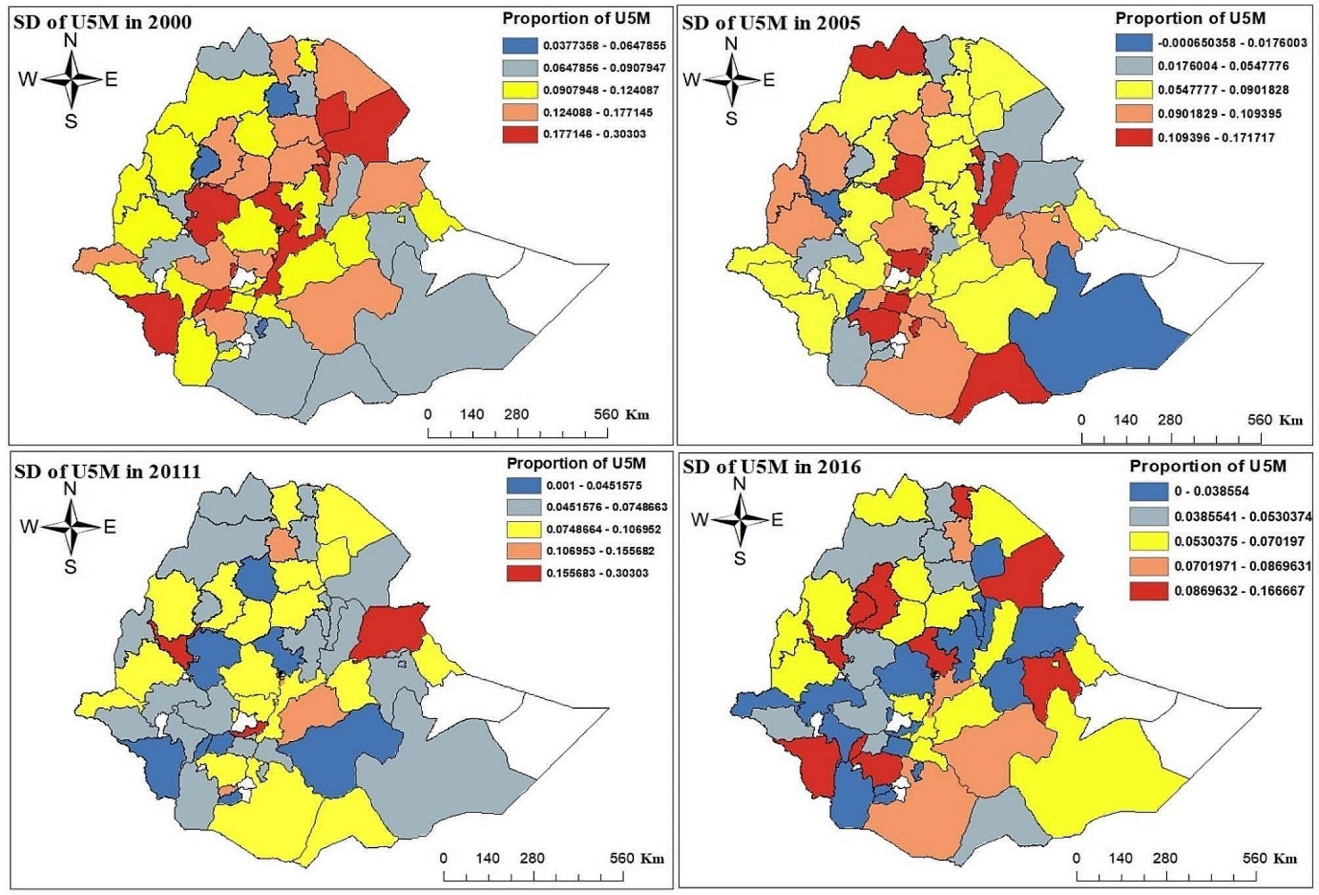
Observed spatial and temporal patterns of under-five mortality in Ethiopian administrative zones

The result of this study showed that the prevalence of under-five mortality in Ethiopia was 12.08%, 8.71%, 7.26%, and 6% for the four EDHS survey data from 2000, 2005, 2011, and 2016 respectively. The result revealed that the prevalence of under-five mortality was decreased at a small rate over five years. In all EDHS surveys, Ethiopian zones had different proportions of under-five child mortality. From 2000 to 2016 EDHS year throughout, the proportion of under-five child mortality observed increased in the North Shewa, Kemash, Awi, Eastern Tigray, Southern Tigray, Mekele, Gamo Gofa, and East Gojjam zones. In 2000 EDHS, Konta Special Woreda, Afar Zone1 had a high proportion of under-five child mortality; this proportion fell in 2005 and 2011 EDHS years; nevertheless, it was once again high in 2016 EDHS year. From 2000 to 2016 EDHS survey year in Jimma, Illubabor, Konso Special Woreda, Zone2, Zone4, and Zone5 in Afar, North Shewa, Zone1, Zone2 in Gambela, and Dire Dawa zone the percentage of under-five child mortality was increased. However, from 2000 to 2016 EDHS year, the rate of under-five child mortality was consistently observed in Jijiga and Hundene zones. The spatial and temporal patterns of under-five mortality are differences in pace overtime at the zonal level (Fig. 3).

**Fig. 4:**
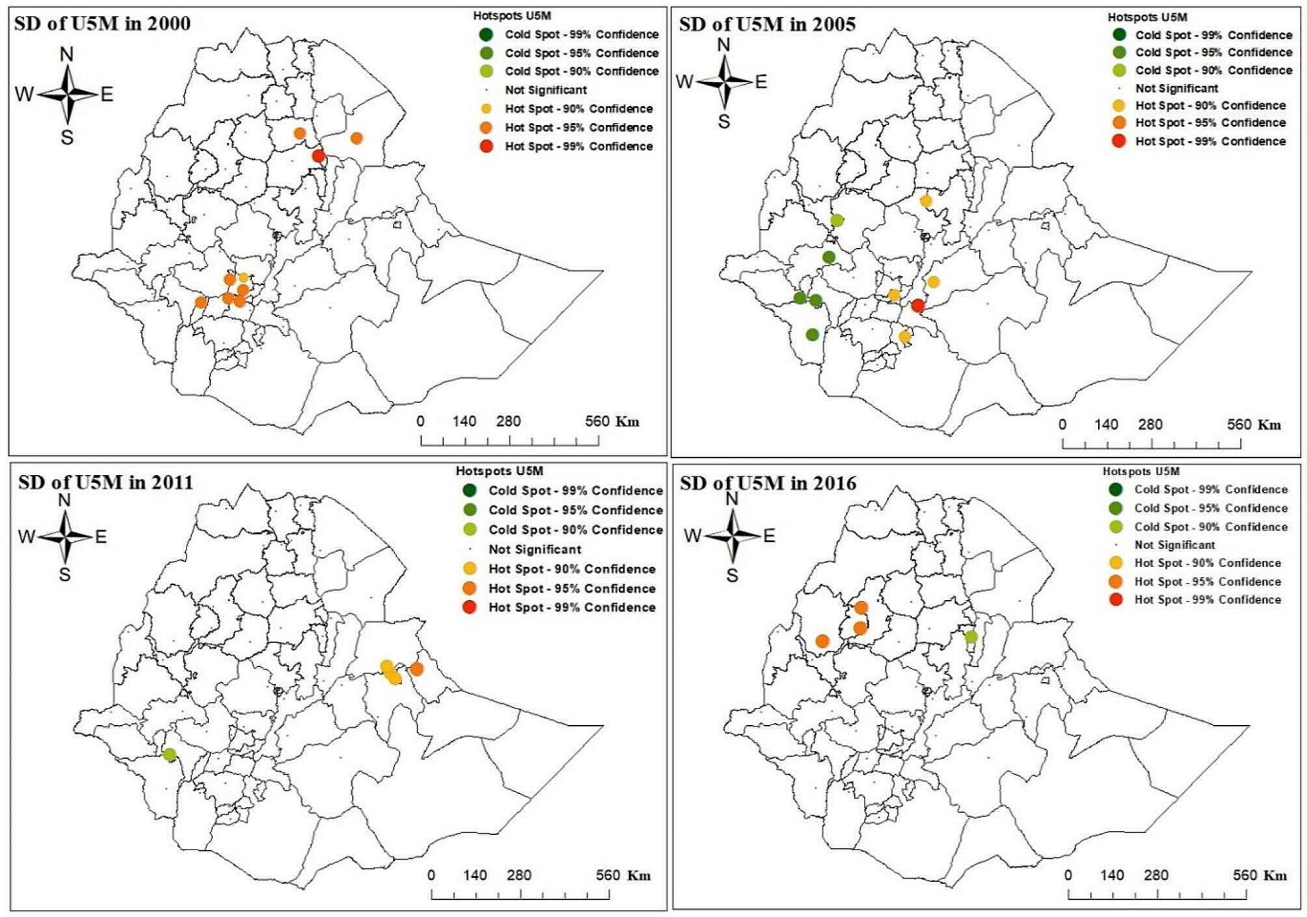
Spatial hotspot analysis in Ethiopian administrative zones across years 2000, 2005, 2011 and 2016

The Hotspot of under-five child mortality at 95% and 99% confidence interval was observed in the zones of Konta-special woreda, yem-special woreda, Gedeo, Hadiya, in SNNP, and Zone 1 in Afar Region, and East shewa, Arsi zones in Oromiya region, and Metekel zone in Benshangul-Gumuz region, North shewa, Awi, Oromiya liyu, and West Gojjam zone in Amhara region are hotspot areas from 2000 to 2016 EDHS year, where the red color indicates significant hot spot (high-risk) and green color indicates the cold spot (low-risk) areas for under-five child mortality at 95% and 99% confidence intervals (Fig. 4).

### Spatial interpolation of under-five mortality

Based on spatial kriging interpolation prediction, the high-risk areas were indicated by red color and the prediction of lower-risk areas was presented by blue color. Zone1, Zone3, and Zone5 in the Afar region, South Wollo, Awi, and South Gonder zone in the Amhara region, Kambata Alamba Tambaro, Shaka, and Hadiya zones in the SNNP Region, Unknowns zone, Shinile zone in the Somalia region, were predicted as more risky zones compared to other zones in 2000–2016 EDHS years.

**Fig. 5:**
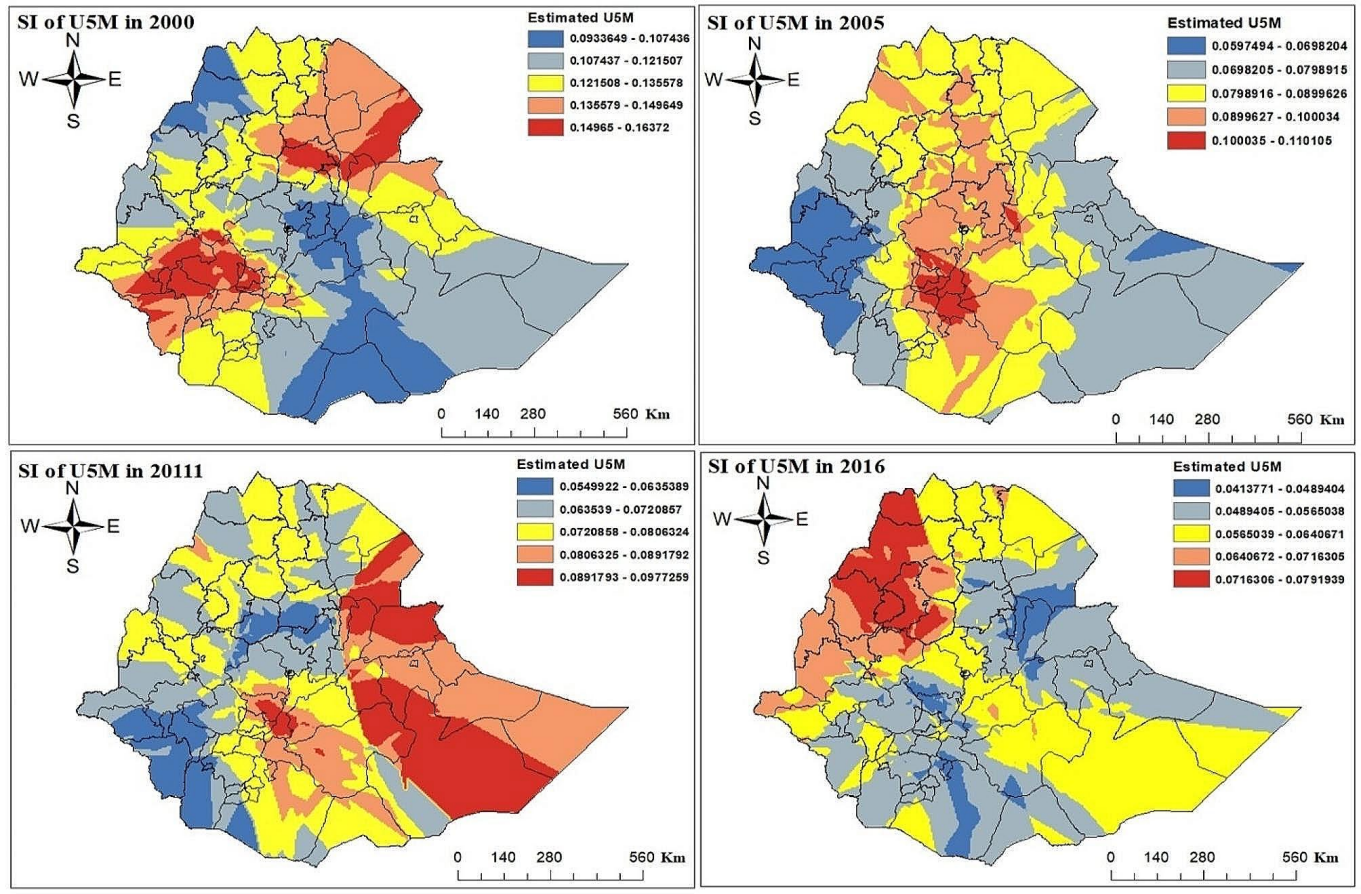
Spatial interpolation of under-five mortality in Ethiopian Administrative zones in 2000–2016

In Zone1, Zone3, and Zone5 in the Afar region, the under-five child mortality rate was high in 2000 and 2011, although it fell in 2005 and the 2016 EDHS year. In Jimma, Shaka, Konta Special Woreda, Benchi-maji, and Hadiya zone the percentage of under-five child mortality fell from 2000 to 2016 EDHS year. However, from 2000 to 2016 EDHS year, the rate of under-five child mortality increased in Awi, East Gojjam, Metekel, and Asosa zones (Fig. 5).

The sill parameter represents the maximum level of spatial dependence in the data, and the nugget parameter represents the variance at zero distance and can be interpreted as the level of measurement error or small-scale variability that is not accounted for in the model. Based on the criteria of sill, nugget, Mean Square Error (MSE), and Root Mean Square Standardized Error (RMSSE), the exponential model in 2005 and 2011, as well as the 2000 and 2016 Gaussian model provides the best fit to the data. It has the lowest nugget value and relatively low MSE and RMSSE values, indicating a good fit for the data. From the two results, the spatial dependence of under-five mortality in the zone is strong, as indicated by the high sill values 0.02763, 0.02245, 0.01434, and 0.02845 from the 2000–2016 EDHS year. However, there is also a significant amount of small-scale variability and measurement error, as indicated by the low nugget values 0.002387, 0.0077, 0.0011, and 0.006 from the 2000–2016 EDHS year. The low MSE (0.04558, 0.03468, 0.0474, 0.03529) and RMSSE (0.8457, 0.94226, 0.9406, 0.97405) values from the 2000–2016 EDHS year indicate that the model (exponential and Gaussian) provides a good fit to the data and can accurately predict the under-five mortality in the levels of zones at unsampled locations(Table 2).

**Table 2:** Characteristics of the semivariogram model

Model	parameter	2000	2005	2011	2016
Exponential	Nugget	0.002274	0.0077	0.0011	0.006
	Sill	0.02453	0. 02245	0. 01434	0.04348
	MSE	0.04765	0.03468	0.0474	0.03549
	RMSSE	0.8597	0.94226	0.9406	0.9949
Spherical	Nugget	0.00228	0.00798	0.0011	0.006
	Sill	0.02844	0. 02000	0.01245	0.01647
	MSE	0.04567	0.03478	0.0469	0.03467
	RMSSE	0.8567	0.95227	0.9482	0.9782
Circular	Nugget	0.00229	0.008	0.0011	0.006
	Sill	0.01268	0.02000	0.01275	0.01458
	MSE	0.04673	0.0348	0.0469	0.03438
	RMSSE	0.85576	0.9549	0.9468	0.97405
Gaussian	Nugget	0.002387	0.0078	0.0012	0.006
	Sill	0.02763	0. 02123	0.01134	0.02845
	MSE	0.04558	0.03454	0.0472	0.03529
	RMSSE	0.8457	0.94819	0.9556	0.97405

**Fig. 6:**
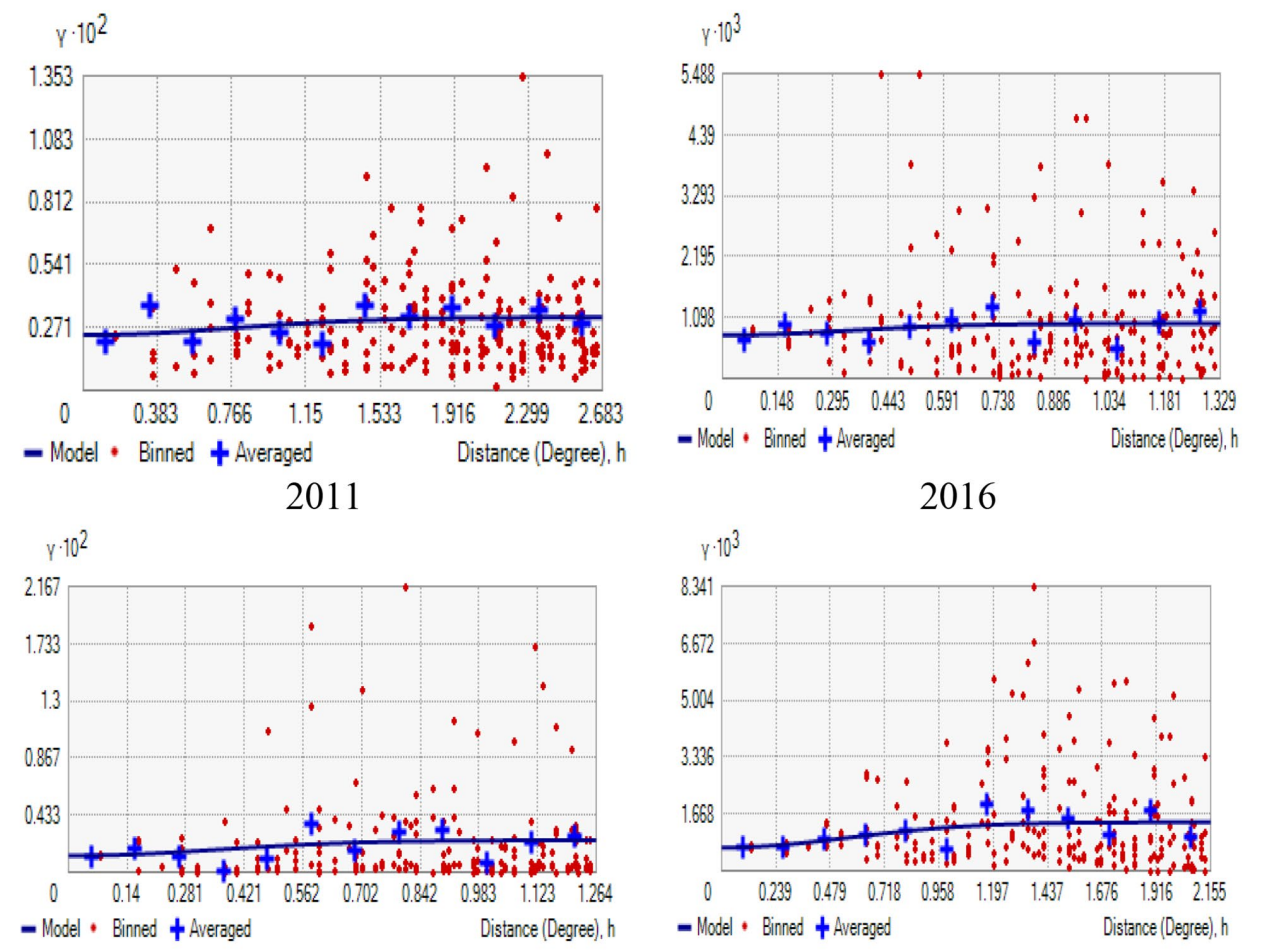
Empirical semi-variogram of under-five mortality in administrative zone

Overall, interpreting the parameters of the selected semivariogram model can provide insights into the spatial structure of the data and it demonstrates that there was significant spatial autocorrelation of the proportion of under-five mortality at zonal level in Ethiopia. This significant autocorrelation confirmed that the space-time dynamic model can be modeled on the assumption of clustering of a child under-five mortality at different zones (Fig. 6).

The four space-time dynamic models of neighborhood settings on U5M of Ethiopian children were compared for model selection. A space-time dynamic general nesting model with the lowest AIC is considered to be the more appropriate model (AIC = 117.919), suggesting that this fitted the data well compared to other models in describing the mortality status of the under-five children (U5M) in administrative zones in Ethiopia (Table 3).

**Table 3:** The model selection criteria for space-time dynamic models

Year	Characteristics	SAC	SDM	SAR	GNSM
2000–2016	Adjusted R^2^	55.965%	53.098%	53.098%	64.746%
	AIC	119.977	160.482	174.147	117.919
	Multiple R^2^	67.918%	65.523%	65.523%	74.160%

The under-five children’s death rate in the previous effect tends to generate a sustained under-five children’s mortality status in the future, according to the substantial and negative temporal dependency (ϕ). For the mortality status of children under the age of five, the effects of the prior year and similar location (γ) as well as the equivalent geographical parameter (ρ) are both statistically significant (*p* < = 0.05). The selected dynamic model identified important confounders related to social, demographic, and health factors.

The spatial-time dynamic model coefficients revealed a negative relationship between zones with a larger percentage of children who were not currently breastfeeding that increased under-five child mortality (β= 0.7685, *p* < 0.001). Higher percentages of illiterate mothers are directly related to increased death rates for children under the age of five (β = 0.4501, *p* < 0.001). Similar to this, the under-five mortality rate of children in the adjoining zone increased by a percentage when the age of first marriage was less than sixteen years old. Additionally, the regression coefficient for antenatal visits is -0.2530, meaning that having more women receive prenatal care at health centers, lowers the mortality rate for children under the age of five in the zones by more than four times. However, the regression coefficients for children in rural areas were positive and substantially spatially dependent, suggesting that the percentage of under-five child mortality in the rural administrative zones was higher than in the urban areas.

The coefficient for the impact of unvaccinated children is 0.9834 which means that for zones with every percentage increase child who was not vaccinated, the under-five child mortality increases by 0.9834. While, the coefficient of the effect of a family with birth order of child is 0.1337, which implies that the percentage of birth order increases the under-five child mortality by 0.1337. The increase in the percentage of access to unimproved source water in the zones was associated with increased under-five mortality of children in the zones by 0.5801, and the increase in the percentage use of unimproved toilet facilities by 0.1385, was associated with increased under-five mortality of children in the zones. Similarly, the increase in the percentage of unemployment status of women was associated with increased under-five mortality of children in the zones by 0.3688.

Furthermore, the spatial and temporal autoregressive coefficients were statistically significant ( ρ and ϕ ) implying that they may also somewhat buffer the effects of the covariates. Despite being negative and smaller in absolute value, the time-space general nesting coefficient is significant. The under-five child mortality rate in a zone is highly influenced by the under-five child mortality rate in the same zone one year before, according to the coefficient of temporal dependence, which is 0.2793. This suggests that the data exhibit strong temporal autocorrelation. The present under-five child mortality in a zone is influenced by the under-five child mortality in that zone’s adjoining zone, according to the geographical dependence value of 0.0406, which indicates that the under-five child mortality at one zone is related to the under-five child mortality at a nearby zone (Table 4).

**Table 4:** Parameter estimation with 95% CI for space-time dynamic models for U5M

variables	GNSM		95% CI for β		95% CI for θ	
	β	Xlag(θ)	Lower CI	Upper CI	Lower CI	Upper CI
Father with illiteracy	-0.3655***	0.7401***	-0.5679,	-0.1629	0.5353	0.9447
Unemployment status	0.3688***	0.8336***	0.1726	0.5649	0.6382	0.9028
Rular child	0.2182*	0.8485**	0.0158	0.4204	0.6436	0.9053
Family with wealth status	-0.0879	0.0731	-0.2826	0.1067	-0.1213	0.2676
Unimproved source water	0.5801***	0.1356	0.3793	0.7808	-0.6261	0.3337
Unimproved toilet facility	0.1890.	0.1385***	-0.0080	0.3861	0.1185	0.1585
Women media exposures	-0.0352	-0.0026	-0.2350	0.1645	-0.2001	0.1948
Mother with illiteracy	0.4501***	-0.0078	0.2442	0.6559	-0.2138	0.1983
Male child	0.0345	0.6265***	-0.1584	0.2274	0.4334	0.8194
Age at first marriage	-0.0281	0.2530**	-0.2284	0.1723	0.2332	0.2727
Family with household sizes	-0.0765	-0.0681	-0.2711	0.1182	-0.2613	0.1253
Birth order of child	0.1159***	0.1137***	0.1054	0.1639	0.1131	0.1531
Marital status with married	-0.3696**	0.2219*	-0.3889	-0.3503	0.2089	0.3149
Preceding birth interval	0.3367***	0.2614**	0.1414	0.5321	0.2419	0.2808
Religions with Christian	0.0711	-0.1149**	-0.1315	0.2736	-0.1345	-0.0953
Mean max temperature	-0.0006	-0.0223	-0.1296	0.1284	-0.1499	0.1053
Mean wet days	0.0033	0.0197	-0.1421	0.1428	-0.1239	0.1632
Mean annual precipitation	0.0277	0.0026	-0.1316	0.0137	-0.0146	0.0197
Mean malaria prevalence	0.0003	0.0008	-0.00006	0.00002	-0.0001	0.0002
Women with contra use	-0.6609***	-0.2942**	-0.8636	-0.4582	-0.4907	-0.0977
Current not breastfeeding	-0.1269	0.7685***	-0.3208	0.0668	0.5633	0.9736
Delivery at a health center	0.0672	-0.2212.	-0.1579	0.2924	-0.4497	0.0072
Child was not vaccinated	0.7681***	0.9834***	0.5683	0.9678	0.1182	0.9845
ANC visits at least 4 times	-0.1585***	-0.1144**	-0.1812	-0.1357	-0.9129	-0.1074
**γ** (Space and time interaction)	-0.1444*		-0.6680		-0.1355	
**ρ** (spatial dependence)	0.0406*		0.0402		0.0990	
ɸ (temporal dependence)	-0.2793**		-0.4903		-0.0682	
**λ**(error term dependence)	-0.4078***		-0.6027		-0.2128	

Key: Signif. Codes: ***P < 0.001, **P < 0.01, *P < 0.05, .*P* < 0.1

There are differences in the spatial and temporal effects on under-five mortality at the zonal level, as shown by the fact that the current under-five child mortality at a zone is strongly influenced by the under-five child mortality at that same zone one year ago and that the current under-five child mortality at a zone is influenced by the under-five child mortality at that neighbouring zone is influenced by the space and time interaction effect of 0.1444.

Since the model already contains coefficients for both space and time, an intercept term is often not included in the dynamic space-time model with spatial and time coefficients. A further intercept term would result in perfect collinearity in the model because the estimates represent the intercepts for each place and time period. In other words, the inclusion of an intercept term would suggest that the intercept is the same for all places and time periods, which is not the case in a dynamic space-time model with spatial and temporal estimates. In order to avoid problems with perfect collinearity and to enable the estimates to represent the intercepts for each site and time period, the intercept term is often left out of models.

**Fig. 7:**
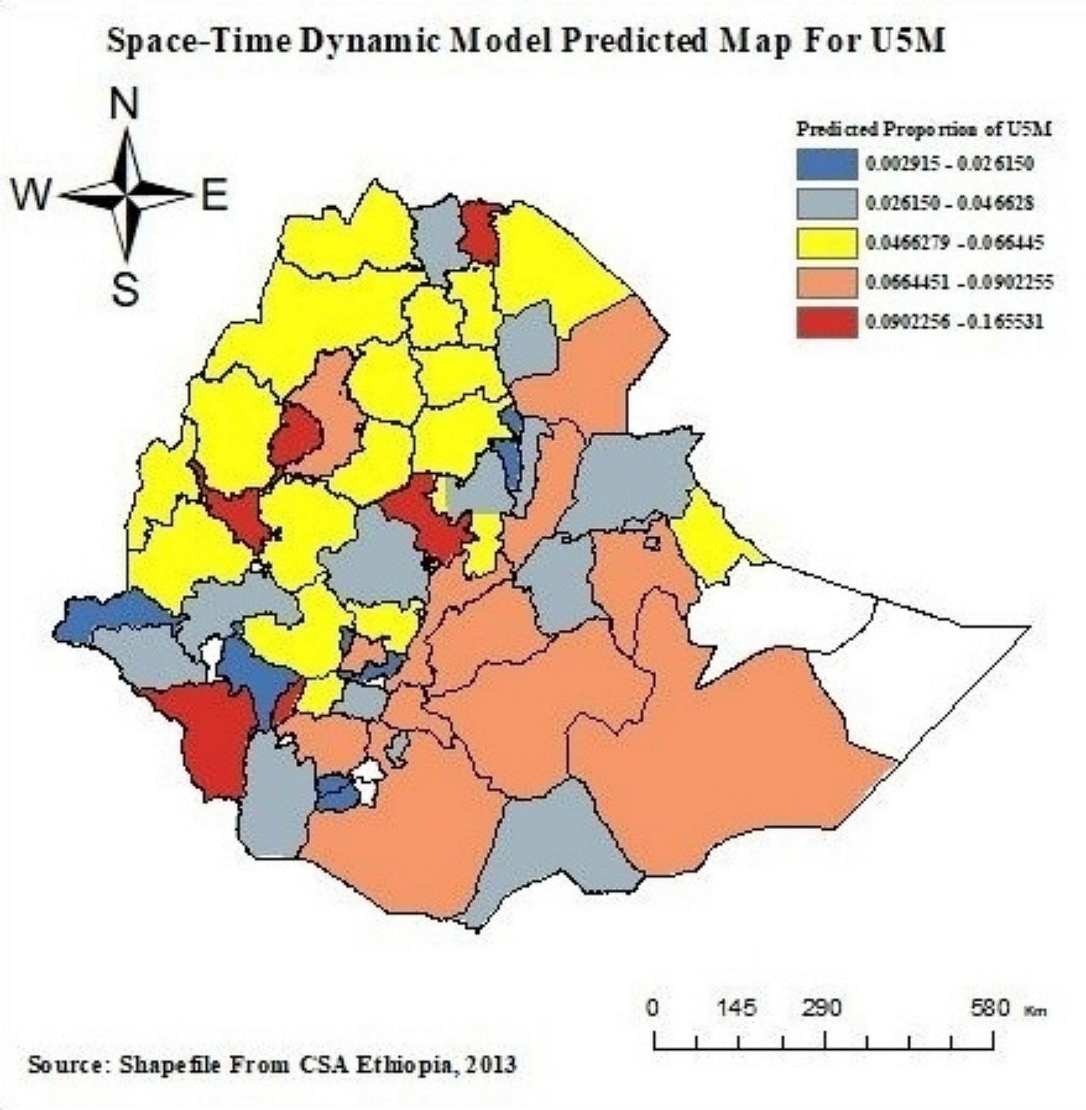
Space-time dynamic general nesting model of U5M in administrative zones

Space-time dynamic prediction of under-five mortality shows that the average proportion of under-five mortality in administrative zones, from those only the Kemash zone in the benshangul gumuz region, Benchi-maji zone in SNNP, and Shinile zone in the Somalia region had a high proportion of under-five mortality at different data waves of observed and predicted distribution in the space-time dynamic general nesting model (Fig. 7).

## Discussion

This study attempted to investigate the spatiotemporal variation of under-five mortality and associated factors in Ethiopia administrative zones based on the EDHS, 2000–2016 dataset which is crucial for informed decision-making, targeted interventions, and monitoring progress in reducing child mortality rates across the country.

This study found that the prevalence of under-five mortality in Ethiopia was 12.08%, 8.71%, 7.26%, and 6% based on data from the EDHS 2000, 2005, 2011, and 2016 respectively. This study’s findings are consistent with those of [19] children who passed away before turning five years. This finding is more significant than that of a study conducted in Bangladesh [20]. The difference in study context and data usage could be the cause.

In order to evaluate the geographic distribution and temporal variation of under-five child mortality (U5M) in the Ethiopian administrative zone using EDHS data from 2000 to 2016, this study revealed a spatial model and space-time dynamics regression analysis technique. This study’s findings are consistent with those of the research [21]. The geographical hot spot study also showed that the SNNP region contains the Konta-special woreda and the Yem-special woreda. This result was in line with one from research done in Ethiopia [22, 23]. This study agreed with the Ethiopian study [12]. Similarly, this research finding demonstrates that East Shewa, Arsi, and North Shewa zones in the Oromia region were high-risk areas for child mortality. This study’s findings were in line with those of a study [24] that was carried out in Ethiopia.

According to our research, male children are more likely than female children to die before the age of five. A similar claim was made in a study conducted in Nigeria and Kenya [25, 26], which discovered that male children had a higher risk of under-five mortality than female children. The study demonstrates that breastfeeding rates are not rising; rather, the percentage of children under the age of five who die is much higher. This study’s findings are consistent with those of earlier ones [25, 27]. The findings of this study are comparable to those of a study conducted in Ethiopia and the Island [28], which found a substantial association between children who were not being breastfed and an increased risk of under-five mortality. As the birth order gets higher, the mortality rate for children under five rises as well. This result was in line with findings from other research done in Kenya, Nigeria, and Ethiopia [29–31]. The mortality of children under the age of five is significantly negatively correlated with the birth interval. Less under-five child mortality occurs in children born four years and more following the previous birth. This research was in line with study [30].

The findings of this study also demonstrated a substantial correlation between the source of unimproved drinking water and under-five child mortality. Under-five child mortality was a risk for children born into a family with an unimproved supply of drinking water. This result was in line with a study [19] that revealed a greater risk of under-five mortality in children who drink water from unsafe sources. Similar to this study, children who drink unimproved water have a significant risk of dying [9]. Additionally, our research indicates a link between rising rates of under-five child mortality and access to unimproved sources of water. This study agrees with the findings of [32].

Our research found a strong correlation between maternal education and under-five child mortality. As a result, a large proportion of under-five child deaths are caused by illiterate women. Similar claims were made in research by [33] that discovered a lesser impact on child survival. Similar to this study, a study conducted in Kenya revealed that the level of a mother’s education greatly affected the mortality rate for children under five [34]. This study showed that the likelihood of a child dying increases with the mother’s level of literacy, and that child mortality rates were lower in households with fewer people. This result is consistent with the result by Yemane et al. [35].

Moreover, the findings of this study indicated a significant and positive relationship between the share of household size and mortality among children under the age of five. In accordance with a study conducted in Ethiopia, this study [36, 37] discovered that as family sizes grow, so does the chance of under-five mortality. The same study found a strong correlation between maternal education and under-five mortality. As a result, a substantial proportion of under-five deaths among women are illiterate. A similar claim was made in a study by [33, 38, 39] that discovered a lesser impact on child survival. Similar to our study, a study conducted in Kenya revealed that the level of a mother’s education greatly affected the mortality rate for children under the age of five [34]. The likelihood of a child dying increases with the mother’s level of literacy, and child mortality rates are lower in households with fewer people. This result is consistent with the results from [35, 40].

Furthermore, this study found a substantial correlation between under-five child mortality and the average effect of first marriage age, religion, marital status, and father education. This result is consistent with other research carried out by several academics in underdeveloped nations like Ethiopia, Nigeria, and Bangladesh [8, 9, 41–43]. According to the same study’s findings, there was a substantial correlation between the percentage of pregnant women who visited a health center more than four times and the under-five child mortality in the zones. The outcome is consistent with the conclusion of [25]. The average direct consequence of a child not receiving vaccinations is an increase of 1% in nearby zones, which over time would result in an increase in under-five child mortality for that zone. The research from [27] lends credence to this conclusion.

## Conclusions

In Ethiopia’s administrative zones, the mortality rate for children under five has been steadily dropping over the four EDHS years of data from 2000 to 2016. This study concluded from the result that there were substantial variations in Ethiopia’s administrative zones between the spatial variation and temporal trends of under-five child mortality. The research investigated the Konta-special woreda, yem-special woreda, Dawuro zone, Kambata Alaba Tambaro, Hadiya, Gedeo, and Guraghe zone, Oromia liyu zone, Awi zone, East Gojjam zone, North Shewa, East Shewa, East Wallega zone, zone1, zone5, Shinile, Kemeshi, and Eastern Tigray zones were high-risk zones of under-five mortality. The space-time dynamic general nesting model is better suited to describing the dependent nature of under-five mortality (U5M) in administrative zones across time. Zone 1 and Zone 5 in Afar Region, North Shewa, East Shewa, Arsi in Oromiya region, and North Shewa zones, Awi Zone, and East Gojjam zone in Amhara region are high-risk areas of child mortality across EDHS years. It revealed that the following risk factors were significant: child not immunised, mother’s illiteracy, unemployment status, preceding birth interval month, father’s illiteracy, ANC visit at least four times, a child was rural, a child was male, use of a contraceptive method, marital status was married. Furthermore, the conclusion is that making it easier for rural communities to access better drinking water close to their houses contributes to meeting the sustainable development goals and reducing the burden of under-five child mortality.

## Data Availability

The dataset used and analyzed during the current study is openly available from EDHS website (https://dhsprogram.com).
